# Phytochemical analysis and anthelmintic activity of *Combretum mucronatum* leaf extract against infective larvae of soil-transmitted helminths including ruminant gastrointestinal nematodes

**DOI:** 10.1186/s13071-024-06194-9

**Published:** 2024-03-01

**Authors:** François Ngnodandi Belga, Patrick Waindok, Marie-Kristin Raulf, Jonathan Jato, Emmanuel Orman, Steffen Rehbein, Verena Spiegler, Eva Liebau, Andreas Hensel, Dieudonné Ndjonka, Christina Strube

**Affiliations:** 1 Faculty of Sciences, Department of Biological Sciences, University of Ngaoundere, P. O. Box 454, Ngaoundere, Cameroon; 2 Institute for Parasitology, Centre for Infection Medicine, University of Veterinary Medicine Hannover, Buenteweg 17, 30559 Hannover, Germany; 3 School of Pharmacy, University of Health and Allied Sciences, PMB 31, Ho, Ghana; 4 Boehringer Ingelheim Vetmedica GmbH, Kathrinenhof Research Center, 83101 Rohrdorf, Germany; 5 Institute of Pharmaceutical Biology and Phytochemistry, University of Münster, Corrensstrasse 48, 48149 Muenster, Germany; 6 Institute of Biology and Plant Biotechnology, University of Münster, Schlossgarten 3, 48149 Muenster, Germany

**Keywords:** Gastrointestinal nematodes, Soil transmitted helminths, *Combretum mucronatum*, Plant extract, Proanthocyanidins, Catechin, Epicatechin

## Abstract

**Background:**

Soil-transmitted helminths (STH) infect more than a quarter of the world’s human population. In the absence of vaccines for most animal and human gastrointestinal nematodes (GIN), treatment of infections primarily relies on anthelmintic drugs, while resistance is a growing threat. Therefore, there is a need to find alternatives to current anthelmintic drugs, especially those with novel modes of action. The present work aimed to study the composition and anthelmintic activity of *Combretum mucronatum* leaf extract (CMLE) by phytochemical analysis and larval migration inhibition assays, respectively.

**Methods:**

*Combretum mucronatum* leaves were defatted with petroleum ether and the residue was extracted by ethanol/water (1/1) followed by freeze-drying. The proanthocyanidins and flavonoids were characterized by thin layer chromatography (TLC) and ultra-high performance liquid chromatography (UPLC). To evaluate the inhibitory activity of this extract, larval migration assays with STH and GIN were performed. For this purpose, infective larvae of the helminths were, if necessary, exsheathed (*Ancylostoma caninum*, GIN) and incubated with different concentrations of CMLE.

**Results:**

CMLE was found to be rich in flavonoids and proanthocyanidins; catechin and epicatechin were therefore quantified for standardization of the extract. Data indicate that CMLE had a significant effect on larval migration. The effect was dose-dependent and higher concentrations (1000 µg/mL) exerted significantly higher larvicidal effect (*P* < 0.001) compared with the negative control (1% dimethyl sulfoxide, DMSO) and lower concentrations (≤ 100 µg/ml). Infective larvae of *Ascaris suum* [half-maximal inhibitory concentration (IC_50_) = 5.5 µg/mL], *Trichuris suis* (IC_50_ = 7.4 µg/mL), and *A. caninum* (IC_50_ = 18.9 µg/mL) were more sensitive to CMLE than that of *Toxocara canis* (IC_50_ = 310.0 µg/mL), while infective larvae of *Toxocara cati* were largely unaffected (IC_50_ > 1000 µg/mL). Likewise, CMLE was active against most infective larvae of soil-transmitted ruminant GIN, except for *Cooperia punctata*. *Trichostrongylus colubriformis* was most sensitive to CMLE (IC_50_ = 2.1 µg/mL) followed by *Cooperia oncophora* (IC_50_ = 27.6 µg/mL), *Ostertagia ostertagi* (IC_50_ = 48.5 µg/mL), *Trichostrongylus axei* (IC_50_ = 54.7 µg/mL), *Haemonchus contortus* (IC_50_ = 145.6 µg/mL), and *Cooperia curticei* (IC_50_ = 156.6 µg/mL).

**Conclusions:**

These results indicate that CMLE exhibits promising anthelmintic properties against infective larvae of a large variety of soil-transmitted nematodes.

**Graphical Abstract:**

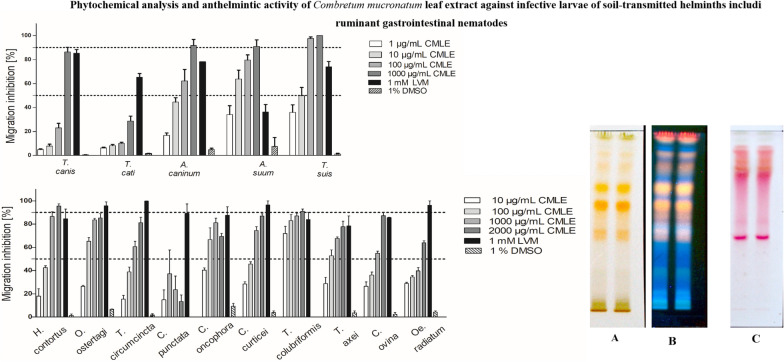

## Background

Soil-transmitted helminth (STH) infections are among the most common parasitic infections in the world and affect more than a quarter of the world’s human population, most of all poor social strata in (sub)tropical regions. The most common STH infecting humans are *Ascaris lumbricoides*, *Trichuris trichiura*, and *Ancylostoma duodenale*, with approximately 1 billion children requiring preventive chemotherapy in 2021 [1]. In addition, several STH of veterinary importance are zoonotic agents known to develop, temporarily become established, or persist in humans. Among them are *Ascaris suum* originating from pigs [2] and *Ancylostoma caninum* [3] and *Toxocara* spp. originating from canids and felids [4]. Nematode of the genera *Ascaris* and *Trichuris* cause the most common helminth infections in humans and pigs worldwide [5]. In both cases, the worms that infect pigs and humans are closely related and difficult to distinguish morphologically [6]. The somatic migration of *A. suum* larvae in pigs results in significant liver condemnation [7]. *Ascaris suum* and possibly *Trichuris suis* are zoonotic parasites [2] and are closely related to *A. lumbricoides* and *T. trichiura*, which infect more than 1 billion and 464.6 million people worldwide, respectively [8, 9]. The hookworm *A. caninum* and the ascarids *Toxocara canis* and *Toxocara cati* are important gastrointestinal parasites of dogs and cats as they may cause enteritis, dehydration, inappetence, despondency, diarrhea, emaciation, weight loss, and can even lead to death in severe cases [10, 11].

Soil-transmitted gastrointestinal nematodes (GINs) are also a major threat to livestock including sheep, goat, and other ruminants [12, 13]. GINs of the family Trichostrongylidae (*Haemonchus*, *Teladorsagia*, *Trichostrongylus*, *Cooperia, Ostertagia*) and Chabertiidae (*Chabertia*, *Oesophagostomum*) are the causative agents of parasitic gastroenteritis, which is one of the primary imperatives to ruminant production [14, 15] as it can result in weight loss, diarrhea, dehydration, anemia, reduced milk production, and reproductive changes [13].

Because of the absence of vaccines for most helminth infections, a primary means of controlling both human and livestock helminths relies almost entirely on synthetic anthelmintic drugs. However, overuse of anthelmintic drugs has led to worldwide emergence of anthelmintic resistance [16–18]. The development of anthelmintic resistance in zoonotic helminths will affect the number of drug options given to humans, which will become increasingly limited [19]. Due to chemoresistance, there is a great effort to investigate novel approaches for solving the problem of anthelmintic resistance by screening traditional medicinal and potential tropical plants with high amounts of bioactive compounds [20–22].

One potential way to develop cheaper and effective anthelmintic drugs is to study indigenous herbal remedies [23]. Evaluation of the activity of traditionally used medicinal plants with documented anthelmintic properties is gaining increasing attention. Several reports, mainly from the African continent, indicate the efficacy of plant extracts against helminth infections in animals [24–26]. The traditional use of medicinal plants in veterinary medicine has been practiced for a long time due to the variable availability and affordability of synthetic anthelmintics, especially in rural areas [19].

*Combretum mucronatum* Schumach & Thonn (Combretaceae) is a forest liane widespread in tropical regions, particularly in the African Savannah forest [27]. In Cameroon, it is used to treat malaria and helminthiasis [28], as well as wounds in other parts of Western Africa [20]. Strong anthelmintic activity against *Trichuris muris* has been documented [29]. Spiegler et al. [30] identified epicatechin, oligomeric procyanidins, and flavonoids as the main compounds of *C. mucronatum* leaf extract and found the procyanidins and the crude extract to be active against the model organism *Caenorhabditis elegans* [30]. However, investigations on anthelmintic activities of *C. mucronatum* against parasites of domestic animals and humans are not available. Therefore, the current work aimed to investigate the phytochemical composition and the anthelmintic activity of *C. mucronatum* leaf extract by thin layer chromatography (TLC) and ultra-performance liquid chromatography (UPLC) as well as larval migration inhibition assays, respectively.

## Methods

### Collection of plant material and preparation of the extract

Leaves of *Combretum mucronatum* were collected in January 2021 in Ngaoundere, Adamawa region of Cameroon (North Latitude of 7°85′88.57" and East Longitude of 13°59′39.19"). The sample was identified by Prof. Tchobsala, a botanist from the University of Maroua (Cameroon). The specimens of *C. mucronatum* were authenticated and a reference sample was deposited at the National Herbarium in Yaoundé, Cameroon under code no. 32193 HNC. The remaining plant material was washed with water, shade-dried at room temperature, and subsequently grounded to powder.

Hydroethanolic extracts were prepared as described previously [30]. Briefly, powdered *C. mucronatum* leaves (1 kg) were defatted with petroleum ether by Soxhlet extraction. The residue was extracted by ethanol:water (1:1 v/v) by use of rotor stator extractor (Ultra-Turrax, IKA-Werke GmbH & Co. KG, Staufen, Germany) at 7500 rpm for 10 min under ice cooling. This extraction was repeated three times and the resulting mixture was filtered with filter paper (Schleicher & Schuell GmbH, Dassel, Germany) under vacuum. The clear solutions were combined and concentrated under vacuum (Rotavapor R-210, Büchi, Flawil, Switzerland) at 40 °C. The obtained aqueous solution was frozen at −20 °C and lyophilized (Alpha 1–4 LD plus Christ, Osterode, Germany). The dried CMLE was stored at 4 °C until further use.

### Thin layer chromatography (TLC)

TLC is a simple and fast technique that allows for the rapid identification of classes of natural products and the ascertainment of the number of compounds present in complex mixtures such as plant extracts [31]. CMLE was dissolved in methanol (MeOH) to a concentration of 10 mg/mL, and 10 µL of the solution were spotted onto silica gel 60 F254 plates (Merck KGaA, Darmstadt, Germany) with an autosampler (CAMAG, Muttenz, Switzerland). The spotted plates were placed into a glass TLC chamber (saturated solvents for 20 min) and developed over a distance of 8 cm in different mobile phases. For flavonoid determination, ethyl acetate:water:formic acid (75:15:5, v:v:v) was used as mobile phase and plates were sprayed with Naturstoff reagent (1% (w/v) diphenylboryloxyethylamine (Carl Roth, Karlsruhe, Germany) for detection. Acetonitrile:water:formic acid (30:8:2, v:v:v) was used as mobile phase to separate proanthocyanidins, which were visualized by spraying the plates with vanillin/hydrochloric acid reagent (1% w/v vanillin in MeOH, followed by addition of 8% Hydrochloric acid (HCl) in glacial acetic acid) and heating to approximately 100 °C. Detection was performed at *λ* = 254 nm, 366 nm, and white light using a CAMAG TLC Visualiser II (visionCATS software, version 3.0) before and after spraying with the respective detection reagents.

### Standardization of CMLE by Ultra High Performance Liquid Chromatography (UPLC)

Analytical UPLC was performed according to a validated method for *C. mucronatum* [32] on an ACQUITY UPLC system with High Strenght Silica (HSS) T3 1.8 µm, 2.1 × 100 mm column; PDA λe detector (λ 210–400); QDa mass-selective detector (positive scan 100.00–1000 Da); autosampler, in-line degasser, and run by Waters Empower 3 software (Waters, Milford, MA, USA). A binary gradient of mobile phase A (containing 0.1% formic acid in water) and B (containing 0.1% formic acid in acetonitrile) was established as follows: t_0min_ A 98%, t_1min_ A 90%, t_2min_ A 90%, t_4min_ A 85%, t_10min_ A 85%, t_11min_ A 0%, t_12min_ A 98%, t_13min_ A 98%. Column temperature: 40 °C; injection volume: 2 µL; flow rate: 0.5 mL/min. Reference standards for external calibration: epicatechin, catechin (Sigma-Aldrich, Taufkirchen, Germany), and procyanidin C1 (isolated and identified as described previously [30]). Reference solutions of epicatechin, catechin, and procyanidin C1 were made by dissolving reference compounds in methanol at concentrations of 10, 25, 50, 100, and 400 µg/mL. CLME was dissolved in methanol at a concentration of 5 mg/mL. A total of five samples of the extract (S1 to S5) were prepared. Prior to injection, all samples were centrifuged at 6000 × *g* (Mikro 120, Hettich Germany) and supernatants were transferred to vials for further analysis to quantify procyanidin C1 and its monomers for standardization. The linear regression equations were *Y* = 1996.4*X* − 2551.9 (*R*^2^ = 0.9983) and *Y* = 2823.6*X* − 29,397 (*R*^2^ = 1) for catechin and epicatechin, respectively, where *Y* is the peak area and *X* defines the respective concentration.

### Larvae of narrow sense STH and ruminant GIN for in vitro studies

Infective larvae of ascarids, whipworms, hookworms, and ruminant (tricho-)strongyle GIN were cultivated from eggs collected at an abattoir or isolated from fecal surplus samples of animals carrying experimental nematode infections for parasite maintenance purposes.

#### Ascarids (*Ascaris suum*, *Toxocara canis*, and *Toxocara cati*)

Eggs of *A. suum* were retrieved from adult females isolated from the intestine of pigs at an abattoir, using only eggs excreted within the first day after isolation. Isolation of *T. canis* (field isolate HannoverTcanis2020) and *T. cati* (field isolate HannoverTcati2019) eggs from fecal samples of experimentally infected dogs and cats was performed via sedimentation–flotation technique. Eggs were embryonated in tap water for at least 6 weeks at room temperature (RT) with periodic oxygenation to allow development of infective third-stage larvae (L3) within the eggs.

Hatching of infective L3 of *A. suum*, *T. canis*, and *T. cati* was performed as described by Raulf et al. [33] with slight modifications. Briefly, embryonated eggs were incubated in 10 mL of 12% NaOCl (Carl Roth, Karlsruhe, Germany) for 5 min (*T. canis* and *T. cati*) or 9 min (*A. suum*). The suspension was washed with 500 mL of sterile phosphate-buffered saline (PBS) over a 10 µm sieve. Eggs were transferred to a 50 mL tube, centrifuged at 1500 × *g* for 5 min, and the pellet was resuspended in 5 mL of culture medium (Roswell Park Memorial Institute media series, Roswell Park Memorial Institute media series (RPMI)-1640 supplemented with 2 mM L-glutamine, 100 U/mL penicillin/100 µg/mL streptomycin, and 250 ng/mL amphotericin B). To promote larval hatching, the eggs were gassed with CO_2_ for 5 min and poured onto 40 µm cell sieves inserted into a 6-well tissue culture plate (Sarstedt, Nümbrecht, Germany) filled with culture medium. Larvae were allowed to migrate for 24–48 h at 37 °C and 5% CO_2_ and were maintained in culture medium until being used in larval migration inhibition assays (LMIA).

#### Whipworms (*Trichuris suis*)

Eggs of *T. suis* (field isolate CopenhagenTs2016) were isolated from fecal samples of experimentally infected pigs via sedimentation–flotation technique and allowed to embryonate to infective first-stage larvae (L1) within the eggs for at least 8 weeks at 37 °C in tap water with periodic oxygenation.

For hatching of infective L1, embryonated eggs were suspended in 10 mL of 3.6% sodium hypochlorite, 0.1 N sulfuric acid solution, and incubated at 37 °C for 45 min. The suspension was washed six times in sterile PBS followed by centrifugation at 800 × *g* for 10 min after every washing step. The eggs were resuspended in culture medium and transferred to 6-well plates [33]. Larvae hatched within 2 days of incubation at 37 °C and 5% CO_2_ and were separated from eggshells by migration through a 15 µm sieve for 2 h before being used in LMIA.

#### Hookworms (*Ancylostoma caninum*)

Feces of dogs experimentally infected with *A. caninum* (field isolate CostaRicaAc2009) were coprocultured for 7–10 days at 26 °C and 70% humidity for development of infective L3. Larvae were retrieved from feces by Baermann–Wetzel technique and stored at 4 °C until further use.

Infective L3 of *A. caninum* were artificially exsheathed according to a method described by Coles et al. [34]. Briefly, larvae were incubated at room temperature with 0.12% of sodium hypochlorite in PBS for 6 min followed by washing with sterile PBS over a 10 µm sieve. The larvae were transferred to a 15 mL falcon tube and left to rest at 37 °C for 2 h. L3 were resuspended in culture medium and maintained until being used in LMIA. Ensheathed infective L3 of *A. caninum* were also used in LMIA.

### Ruminant gastrointestinal nematodes (GIN)

Feces of ruminants experimentally infected with recent field isolates as per the International Cooperation on Harmonisation of Technical Requirements for Registration of Veterinary Medicinal Products (VICH) guidelines [35] (sheep: *Haemonchus contortus*, *Teladorsagia circumcincta, Trichostrongylus colubriformis*, *Trichostrongylus axei*, *Chabertia ovina, Cooperia curticei*; cattle: *Cooperia punctata*, *Cooperia oncophora*, *Ostertagia ostertagi*, *Oesophagostomum radiatum*) were coprocultured for 7–10 days at ~ 26 °C for development of infective L3. The L3 were retrieved from the coproculture by the agar layer technique and stored at 4 ~ 8 °C until further use.

Infective L3 were exsheathed as described by Coles et al. [34] with slight modifications. Briefly, larvae were incubated at room temperature with 0.2% sodium hypochlorite in PBS for 6–9 min. Afterward, larvae were rinsed with PBS over a 15 µm sieve and left for rest in PBS at 4 °C overnight. Exsheathed L3 were placed on 20 µm or 25 µm sieves for migration for 2 h at 37 °C before being used in LMIA.

### Larval migration inhibition assays (LMIA)

For each assay, a stock solution of the hydroethanolic CMLE was freshly prepared by dissolving the extract in DMSO by vigorous vortexing, followed by adjusting the concentration of the dissolved extract to 10 mg/ml in culture medium with a concentration of DMSO not exceeding 1%.

LMIA was performed as described by Demeler et al. [36, 37]. This method uses a migration system that physically separates motile from non-motile larvae through a filter mesh (Sefar Nitex^®^, Sefar Holding AG, Thal, Switzerland). The mesh size allows active larvae, but not immotile larvae, to pass through the mesh. Accordingly, mesh sizes were chosen on the basis of the diameter of larvae of each parasite species.

From stock solutions, four concentrations of extract (1, 10, 100, and 1000 µg/mL for STHs or 10, 100, 1000, and 2000 µg/mL for ruminant GIN) were prepared by serial dilution in culture medium in a 48-well plate to a final volume of 200 µL per well. Levamisole-HCl (purity ≥ 99.0, Glentham Life Sciences, 1 mM, 241 µg/mL in culture medium) served as positive control and DMSO (1% in culture medium) as negative control. A total of 10–20 larvae were pipetted into a well supplemented with the respective treatment solutions and incubated at 37 °C, 5% CO_2_ for 24 h (*T. suis*), 48 h (ruminant GIN), or 72 h (ascarids, *A. caninum*). After incubation, the larvae were transferred to a 48-well migration plate equipped with sieves [37]. The migration plates were incubated at 37 °C, 5% CO_2_ for 24 h. The migration chambers were then lifted and the sieve was inverted to the next well in the near row. Non-migrating larvae adhering to the sieve were washed into the corresponding well with 1000 µL of PBS. Determination of the inhibition of infective larvae migration was performed under a Zeiss ID03 inverted microscope (Carl Zeiss) and the percentage of non-migrating larvae was calculated relative to the total number of larvae per well. All trials were performed in three independent experiments using four technical replicates for each trial.

### Statistical analyses

Data were presented as the average of at least three independent experiments. Data were summarized in Microsoft Excel (Microsoft Office Professional Plus 16). Linear or nonlinear regressions were generated from the data using GraphPad Prism software (version 8.0.1, San Diego, California, USA). One-way analysis of variance (ANOVA) followed by Tukey’s test for multiple comparisons was used to analyze the data. A *P* ≤ 0.05 was considered statistically significant.

## Results

### Phytochemical analysis

**Fig. 1:**
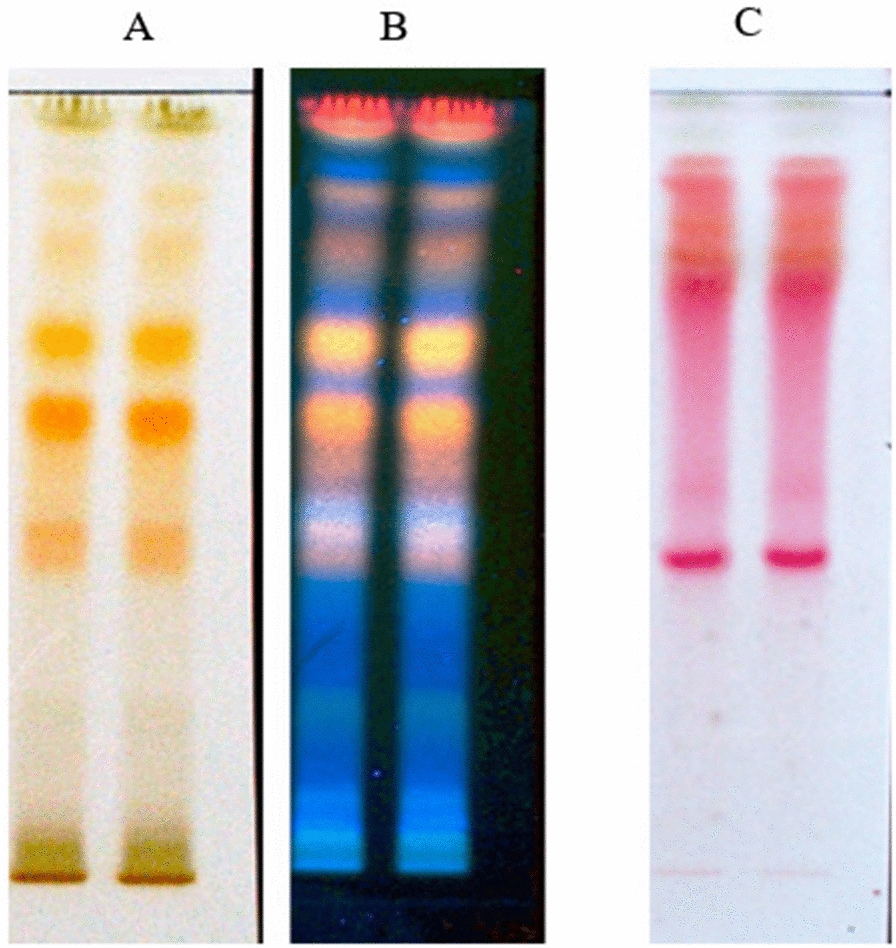
TLC profiles of *Combretum mucronatum* leaf extract CMLE, detected with Naturstoff reagent under white light (**A**) and at *λ* = 366 nm (**B**), or detected with vanillin/HCl (**C**). Flavonoids appear as yellow bands (**A**, **B**), while strong red bands represent proanthocyanidins (**C**)

Prior to the in vitro bioassays, the phytochemical composition of the hydroethanolic extract from the leaves of *C. mucronatum* (CMLE) was characterized by TLC and UPLC. Visualization of flavonoids by use of Naturstoff reagent (diphenylboryloxyethylamine, 1% in MeOH) showed several bands under white light (Fig. 1A) and at *λ* = 366 nm (Fig. 1B) in TLC. Two strong yellow fluorescent bands together with a series of weaker bands in yellow–orange, yellow–green, and blue were observed at *λ* = 366 nm (Fig. 1B), suggesting different types of flavonoids and phenolic compounds. Vanillin reagent revealed the presence of proanthocyanidins in the *C. mucronatum* extract (Fig. 1C).

High performance liquid chromatography coupled with photodiode array (PDA) and mass spectrometry (MS) was used to identify potential marker compounds in CMLE. The trimeric procyanidin C1, as well as the typical flavan-3-ols epicatechin and catechin, were detected in positive ionization mode and at a wavelength of 280 nm. Catechin and epicatechin were quantified in CMLE by UPLC by using catechin and epicatechin standards. The calibration curves were linear over the concentration range of 10–400 µg/mL for catechin and 25–400 µg/mL for epicatechin. Catechin and epicatechin contents were calculated as 16.4 ± 0.6 µg per mg dry extract and 1.0 ± 0.2 µg per mg dry extract, respectively. Procyanidin C1 (*m*/*z* 867 Da) was detected in the extract but not quantified, as the respective peak area was very small. There was no significant difference between the catechin and epicatechin content of five different samples (Table 1). This result shows that the compounds in the *C. mucronatum* extract were uniformly distributed, but also provides the content of the reference compounds as an additional phytochemical characterization.

**Table 1:** Quantification of epicatechin and catechin in *Combretum mucronatum* leaf extract (CMLE)

Samples	Epicatechin (µg/mg)	Catechin (µg/mg)
S1	1.0 ± 0.2	15.2 ± 0.1
S2	1.1 ± 0.2	15.7 ± 1.1
S3	0.8 ± 0.4	17.9 ± 0.6
S4	1.2 ± 0.3	17.2 ± 0.8
S5	0.9 ± 0.2	16.7 ± 0.7
Mean ± SD	1.0 ± 0.2	16.4 ± 0.6

*SD* standard deviation

### In vitro anthelmintic activity of *Combretum mucronatum* against larvae of narrow sense STH

**Fig. 2:**
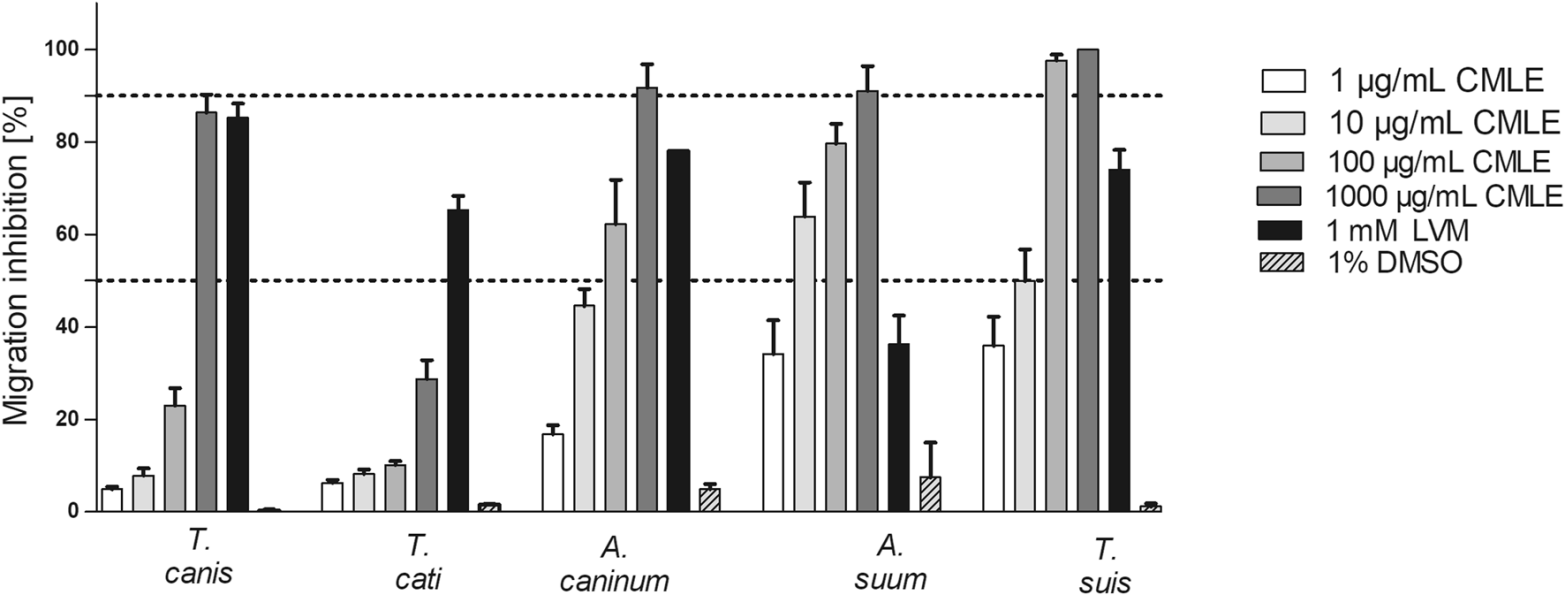
Inhibitory effects of *Combretum mucronatum* leaf extract (CMLE) and levamisole (LVM, 1 mM; positive control) on the larval migration of infective soil-transmitted helminth larvae after 24 h (*Trichuris suis*) or 72 h, respectively. Negative control represents RPMI 1640 containing 1% DMSO. Results are the means of three independent experiments, each performed in four technical replicates. Dotted lines indicate the IC_50_ and IC_90_. Error bars indicate the standard error of the mean (SEM)

A dose-dependent inhibitory effect of CMLE was observed on the migration of most STH infective larvae (Fig. 2). With an IC_50_ of 5.5 (1.4–9.7) µg/mL, hatched infective larvae of *A. suum* were most susceptible to the treatment with CMLE (Table 2). At the highest concentration, inhibition of larval migration was more than 90%, which was significantly higher than the negative control with 7.5% larval migration inhibition (*F*_(5, 11)_ = 30.51, *P* < 0.001). *Ascaris suum* larvae were more sensitive to high concentrations of the *C. mucronatum* extract than to levamisole (1000 µM) with inhibition of larval migration of only 36.3% (*F*_(5, 11)_ = 30.51, *P* = 0.028) (Fig. 2).

**Table 2:** Half-maximal inhibitory concentrations (IC_50_) of *Combretum mucronatum* leaf extract (CMLE) against infective larvae of soil-transmitted helminths

Parasites	IC_50_ (95% CI) (µg/mL)
*Ascaris suum*	5.5 (1.4–9.7)^a^
*Ancylostoma caninum*	18.98 (1.8–36.1)^a^
*Toxocara canis*	310.0 (192.0–428.0)^b^
*Toxocara cati*	> 1000
*Trichuris suis*	7.4 (−7.3 to 22.1)^a^

Small letters compare means. Different letters indicate significantly different values (*P* < *0.05*), *CI* confidence interval for mean

Furthermore, hatched infective *T. suis* larvae showed a high susceptibility to CMLE as indicated by an IC_50_ of 7.4 (−7.3 to 22.1) µg/mL (Table 2). At the highest concentration of CMLE, all of the larvae were non-motile (Fig. 2), whereas only 74.0% of larvae were inhibited by levamisole (*P* > 0.05). Migration inhibition in the negative control was 1.2%, which was significantly lower compared with high concentrations of the CMLE (*F*_(5, 12)_ = 81.89, *P* < 0.001).

With an IC_50_ of 18.9 (1.8–36.1) µg/mL, exsheathed infective larvae of *A. caninum* were also sensitive to the extract (Table 2), while ensheathed infective larvae were not affected by the extract (data not shown). At the highest CMLE concentration, the percentage of migration inhibition was 89.2%, which was significantly higher than that of the negative (1% DMSO) (*F*_(5, 12)_ = 51.77, *P* < 0.001) as well as the positive control (1000 µM, levamisole-HCl) (*F*_(5, 12)_ = 51.77, *P* < 0.001).

Compared with the other STH, inhibition of the migration of *Toxocara* spp. infective larvae occurred only at higher doses. Accordingly, the IC_50_ values were considerably higher, with 310.0 (192.0–428.0) µg/mL for *T. canis* and even more than 1000 µg/mL for *T. cati* (Table 2). Statistical analyses showed that larvae of *T. cati* were less sensitive to CMLE than those of *T. canis* (*F*_(4, 12)_ = 41.53, *P* < 0.001) at the highest concentration (1000 µg/mL), while at lower concentrations, there was no significant difference (95% CI −5 to 2, *P* = 0.59). The highest migration inhibition was observed at 1000 µg/mL with 86.4% of *T. canis* and 28.8% of *T. cati* larvae being non-motile. The percentage of non-migrating larvae was significantly higher than that of the negative control (0.35–1.53%; *F*_(5, 12)_ = 123.3, *P* = 0.02 to *F*_(5, 12)_ = 51.77, *P* < 0.001). The highest concentrations of CMLE had a comparable effect to the positive control (95.8%) (*F*_(1, 8)_ = 0.049, *P* = 0.83) against *T. canis* infective larvae.

### In vitro anthelmintic activity of *Combretum mucronatum* against larvae of ruminant GIN

**Fig. 3:**
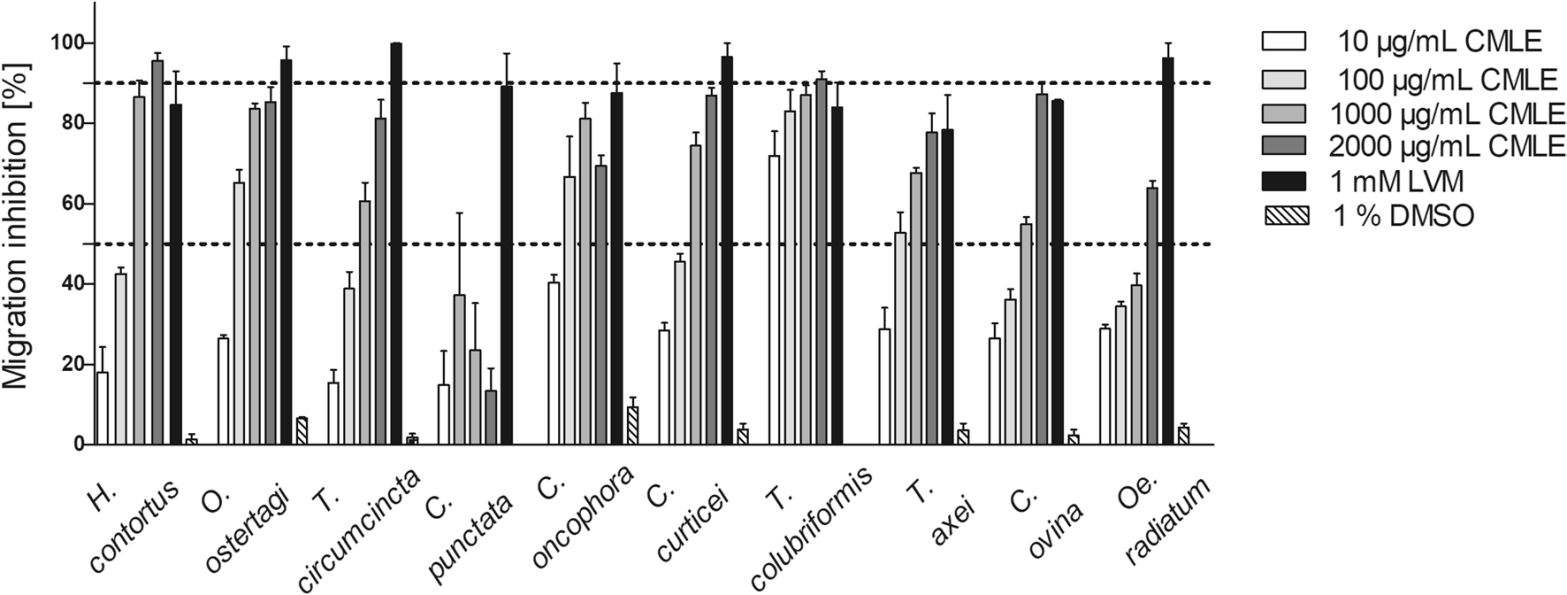
Inhibitory effects of *Combretum mucronatum* leaf extract (CMLE) and levamisole (LVM, 1 mM; positive control) on the larval migration of exsheathed infective third-stage larvae of ruminant gastrointestinal nematodes after 48 h. Negative control represents RPMI 1640 containing 1% DMSO. Results are the means of three independent experiments, each performed in four technical replicates. Dotted lines indicate the IC_50_ and IC_90_. Error bars indicate the standard error of the mean (SEM)

The effect of CMLE on the migration of exsheathed L3 of ruminant (tricho-)strongyles of ruminants was generally dose dependent. In the negative controls, the percentage of migration inhibition ranged from 0.0% to 9.4%. Hence, DMSO at 1% did not impact larval migration significantly, indicating that observed changes in larval migration in the treatment wells could be ascribed to the CMLE. At the highest concentration (2000 µg/mL), CMLE significantly inhibited migration of larvae of *H. contortus* (95.6 ± 3.8%), *T. colubriformis* (90.9 ± 3.6%), *C. ovina* (87.2 ± 5.2%), *C. curticei* (86.9 ± 3.4%), *O. ostertagi* (85.2 ± 6.4%), *T. circumcincta* (81.2 ± 10.4%), *T. axei* (77.7 ± 10.8%), *C. oncophora* (69.4 ± 4.6%), and *Oe. radiatum* (63.9 ± 4.1%). However, CMLE was inactive against *C. punctata* larvae, where the percentage migration inhibition ranged from 13.4% to 37.2%, while 96.4% inhibition resulted in the levamisole positive control (1 mM; Fig. 3). At a concentration of 2000 µg/mL, significant differences were observed between *C. punctata* and all other ruminant GIN (*F*_(9, 28)_ = 11, *P* < 0.01 to 0.001).

## Discussion

The present study indicated that the extract CMLE from *C. mucronatum* typically contains proanthocyanins and flavonoids as main constituents. This result is in agreement with those obtained by Bekoe et al. [38] and Orman et al. [32], who found different flavonoids in the leaf extracts from *C. mucronatum*. Quantification of epicatechin and catechin, representing the monomeric units of the oligomeric procyanidins, was performed by UPLC. Unfortunately, the amount of procyanidin C1 in CMLE was too low for standardization. This result was in contrast to Spiegler et al. [30], who detected a considerable amount of procyanidin C1 in the leaf extract of *C. mucronatum* harvested from Ghana (West Africa). However, TLC analysis showed that CMLE contains the proanthocyanidins with a higher degree of polymerization that were not captured by HPLC. This might be explained by differences in climate, collection period, and harvest area among the studies [32]. The contents of epicatechin and low molecular weight procyanidins were particularly affected by seasonal and spatial variation [32] In addition, these authors have found that tannin, flavonoid, and saponin contents from *Sterculia setigera* and *Sclerocarya birrea* extracts were higher in Kem than those in Yola, two localities in Nigeria [39]. In contrast, epicatechin and catechin were well detectable, with a higher content of catechin (16.4 ± 0.6 µg per mg dry extract) compared with epicatechin (1.0 ± 0.2 µg per mg dry extract).

Larval migration inhibition [37] and adult worm motility [40] tests are widely used in veterinary parasitology for the search for new anthelmintic compounds [41] or the detection of anthelmintic resistance [37]. The advantage of in vitro tests is that the compounds or plant extracts to be tested are in direct contact with the parasite life stages, allowing for initial efficacy screening. Moreover, these tests are relatively inexpensive. Spiegler et al. [30] and Koné et al. [29] previously reported in vitro efficacy of *C. mucronatum* extract against *C. elegans* and *T. muris.* The current study revealed anthelmintic activity of CMLE against a panel of STH including ruminant GIN. The effect of CMLE was generally dose-dependent and varied considerably between parasite species. Regarding narrow sense STH, *A. suum*, *A. caninum*, and *T. suis* were most sensitive to the extract as opposed to *T. canis* and *T. cati*, and striking differences in IC_50_ values against the *Ascaris*, *Trichuris*, and *Ancylostoma* (5.54–18.98 µg/mL) versus the *Toxocara* (310.02–7249.85 µg/mL) species were observed. A similar pattern was reported by Jato et al. [42] when testing a *Phyllanthus urinaria* extract against larvae of these genera. Both *C. mucronatum* and *P. urinaria* are traditional medicinal plants against helminthiases [20], and the substantially lower IC_50_ values against *Ascaris*, *Trichuris*, and *Ancylostoma* infective larvae as compared with *Toxocara* infective larvae might mirror their selection by traditional healers against parasite genera using humans as definitive host. Similarly, Spiegler et al. [43] reported that infective larvae of *Trichuris vulpis* were more sensitive to *Paullinia pinnata* root extract than infective *T. cati* larvae and *C. elegans*, while no effect of the *P. pinnata* extract on egg hatching of *H. contortus* or the motility of infective ensheathed *H. contortus* and ensheathed *A. caninum* larvae was observed. Infective ensheathed *A. caninum* larvae were also tested in the present study, but larval migration was not affected by CMLE (data not shown), compared with a significant migration inhibition of infective exsheathed *A. caninum* larvae. Consequently, ensheated infective STH/GIN larvae seem to be (more or less) protected against (at least some) bioactive plant compounds by their coverings, i.e., cuticle sheath, which also protect the larvae from desiccation and other damage in the environment. After being ingested by a suitable host, infective larvae hatch from the eggs or exsheath in the gastrointestinal tract. This process was artificially induced in the present study, and at this stage, infective larvae of most of the 15 STH/GIN species tested were susceptible to CMLE as indicated by a significant inhibition of larval migration.

As with the narrow sense STH described above, migration inhibition of ruminant GIN infective larvae showed a dose-dependent pattern, except for *C. punctata* larvae, which were insensitive to CMLE. Similarly, Yongwa et al. [40] have shown that *Senna italica* extract has dose-dependent effect on infective L3 of *H. contortus*. Likewise, *Carica papaya* has presented strong activity on infective larvae of *H. contortus* with dose-dependent effect [44]. In the present study, a high anthelmintic activity of the CMLE was observed on exsheathed infective larvae of *T. colubriformis* with IC_50_ values of 2.07 ± 2.98 µg/mL. Likewise, exsheathed infective larvae of *T. axei*, *H. contortus*, *O. ostertagi*, *C. oncophora*, and *C. curticei* have been shown to be sensitive against CMLE with slightly higher IC_50_ values (cf. Table 3). Similar to the narrow sense STH tested, the current results also showed variable anthelmintic efficacies of CLME on different ruminant GIN species. This finding is in accordance with Molan et al. [45], who performed in vitro tests on infective larvae of *H. contortus*, *T. circumcincta*, and *T. colubriformis* and found divergent effects of extracts of sulla (*Hedysarum coronarum*), a legume forage. These authors have shown that *T. colubriformis* larvae were more refractory to the inhibitory effects of *H. coronarum* extract than larvae of the other species. Shepherd et al. [46] evaluated in vitro anthelmintic activity of heather extract on *T. colubriformis* and *T. circumcincta* larvae and found that it was effective against *T. circumcincta*, but not against *T. colubriformis*. Nevertheless, the parasite species as well as the extracted plant species drive differences in larvae mobility inhibition. For example, against *H. contortus,* our study showed a larval migration inhibition rate of more than 80% at 1000 µg/mL, which is stronger than that reported for other tanniferous plant extract against the same parasite by Barrau et al. [47].

**Table 3:** Half-maximal inhibitory concentrations (IC_50_) of *Combretum mucronatum* leaf extract (CMLE) against infective larvae of ruminant gastrointestinal nematodes

Parasites	IC_50_ (95% CI) (µg/mL)
*Chabertia ovina*	349.7 (307.4–391.9)^bc^
*Cooperia curticei*	156.6 (66.4–246.9)^ab^
*Cooperia oncophora*	27.6 (−24.7 to 80.0)^a^
*Cooperia punctata*	n.d.
*Haemonchus contortus*	145.7 (101.7–189.7)^ab^
*Oesophagostomum radiatum*	995.3 (781.7–1208.9)^d^
*Ostertagia ostertagi*	48.5 (−21.4 to 118.4)^a^
*Teladorsagia circumcincta*	470.5 (167.7–772.7)^c^
*Trichostrongylus axei*	54.7 (23.3–86.1)^a^
*Trichostrongylus colubriformis*	2.1 (−5.5 to 8.8)^a^

*n.d.* not determinable

Small letters compare means. Different letters indicate significantly different values (*P* < *0.05*), *CI* confidence interval for mean

It was proposed that the anthelmintic activity of CMLE against *C. elegans* was due to the procyanidin oligomers [30]. The current study showed that *C. mucronatum* harvested in Cameroon contains a low amount of procyanidin C1 as a representative oligomeric procyanidin, but has a good anthelmintic effect against larvae of STH/GIN. As suggested by TLC analysis, the efficacy of CMLE in this work could be due to oligomeric proanthocyanidins with a higher degree of polymerization that were not captured by HPLC. In addition, Greiffer et al. [48] have shown that a *C. mucronatum* leaf extract acted on the cuticle of *C. elegans,* inducing disruptions of the cuticle structure and molting defects at all larval stages. The authors have shown that the tissues of *C. elegans* were not affected by the treatment. Thus, the anthelmintic effect of CMLE found in the present study could be due to its action on the cuticle of helminth larvae. Previous research on the synergistic inhibition of the exsheathment of *H. contortus* infective L3 by flavonoid monomers (luteolin or quercetin) and condensed tannins (proanthocyanidins or prodelphinidins) was shown [49]. Williams et al. [50] reported that procyanidin-rich extracts/fractions may have higher activity against *Oesophagostomum dentatum*, which is somewhat surprising given that prodelphinidins (PDs) are generally considered to have a higher biological effect than procyanidins (PCs) due to an additional hydroxyl group promoting increased hydrogen bonding with proteins [51].

Variation in the anthelmintic property of the extract tested in this study could be ascribed to the different susceptibility of parasite species toward tannins or different compounds present in CMLE. This result is in accordance with those found by Quijada et al. [52], who reported that condensed tannins have different modes of action against different parasite species. Considering their mode of action, anthelmintic drugs may be distinguished into nicotinic agonists, acetylcholinesterase inhibitors, GABA agonists, GluCl potentiators, calcium permeability increasers, β-tubulin binders, proton ionophores, inhibitors of malate metabolism, inhibitors of phosphoglycerate kinase and mutase, inhibitors of arachidonic acid metabolism, and stimulators of innate immunity [53, 54]. Indeed, levamisole acts as a selective nicotinic agonist to induce contraction of nematode somatic muscle tissue and therefore leads to paralysis [55]. The known target sites on nematode parasites are proteins including ion channels, enzymes, structural proteins, and molecule transporters [53, 54]. Consequently, different bioactive compounds of *C. mucronatum* could have different targets to exert their anthelmintic activity on infective larvae. Generally, tannins bind proline-rich proteins with high affinity [56], and are thus believed to interfere with helminth parasites by binding to proline-rich collagenous tissues such as the cuticle [57–59]. Apart from a varying susceptibility toward components of the extract other than tannins, differences in the efficacy of CMLE against the different nematode species could also arise from differences in the cuticle composition. For example, the ratio of hydroxyproline compared with proline has been found to vary among species [60]. On the contrary, polyhydroxyproline binds tannins with a much lower affinity than polyproline [56], which could in part explain the observed differences in activity. In addition, several studies suggest that tannins are not the only principal secondary metabolites responsible for anthelmintic property on GIN, as synergistic effects of condensed tannins with flavonoid monomers were also previously shown [61, 62]. Furthermore, von Son de Fernex et al. [63] recorded a synergistic effect between quercetin and caffeic acid from *Leucaena leucocephala* on egg hatching of *Cooperia* species in their study. It should also be mentioned that substances or compounds that are effective in vitro are not necessarily effective in vivo. With respect to the anthelmintic activity of condensed tannins, extracts of sainfoin (*Onobrychis viciifolia*), for example, showed in vitro activity against different GIN of sheep and cattle [47, 64]. However, in vivo activity was determined as the reduction of fecal egg count was observed against *H. contortus* in lambs that were fed sainfoin [65], but not against *O. ostertagi* and *C. oncophora* in calves receiving the pelleted plant as feed [66]. Therefore, in vivo validation deserves further investigations prior to potential use for helminth control in domestic animals.

## Conclusions

The results obtained in the present study showed that an extract from the leaves of *Combretum mucronatum* possesses anthelmintic activity against infective larvae of different parasitic nematode species. Infective larvae of STH such as *T. suis*, *A. suum*, and *A. caninum* were more sensitive to the CMLE than those of *Toxocara* species. Moreover, CMLE had a significant effect on ruminant GIN larvae, except for *C. punctata*. Regarding its phytochemical composition, different phenolic compounds represented the major class of natural products in the extract. Whether the extract is suitable for the in vivo treatment of helminth infections has to be clarified in further studies.

## Data Availability

Data supporting reported results is contained within the article.

## Abbreviations

CMLE: *Combretum mucronatum* leaf extract
DMSO: Dimethyl sulfoxide
GIN: Gastrointestinal nematode
L1: First stage larvae
L3: Third stage larvae
LMIA: Larval migration inhibition assay
PBS: Phosphate-buffered saline
RPMI: 1640, Roswell Park Memorial Institute media series 1640
STH: Soil-transmitted helminth
TLC: Thin layer chromatography
UPLC: Ultra-high performance liquid chromatography
WHO: World Health Organization

## References

[1] WHO. Soil-Transmitted Helminthiases. Number of children (Pre-SAC and SAC) requiring preventive chemotherapy for Soil-Transmitted Helminthiasis. 2022. https://apps.who.int/neglected_diseases/ntddata/sth/sth.html. Accessed 20 Jun 2023

[2] Nejsum P, Betson M, Bendall RP, Thamsborg SM, Stothard JR (2012). Assessing the zoonotic potential of *Ascaris suum* and *Trichuris suis*: looking to the future from an analysis of the past. J Helminthol.

[3] George S, Levecke B, Kattula D, Velusamy V, Roy S, Geldhof P (2016). Molecular identification of Hookworm isolates in humans, dogs and soil in a Tribal Area in Tamil Nadu, India. PLoS Negl Tropical Dis.

[4] Strube C, Heuer L, Janecek E (2013). *Toxocara* spp. infections in paratenic hosts. Vet Parasitol.

[5] Holland C, Boes J, Holland CV, Kennedy MW (2002). Distribution and predisposition: people and pigs. The geohelminths: ascaris, Trichuris and hookworm. World class parasite.

[6] Muramatsu R, Sato R, Onuma N, Sasai K, Shibahara T, Matsubayashi M (2020). Molecular identification of *Trichuris suis* worms and eggs in pig feces, infected intestines, and farm environments in Japan. JARQ.

[7] Roepstorff A (2003). *Ascaris suum* in pigs: population biology and epidemiology.

[8] Shah J, Shahidullah A (2018). *Ascaris lumbricoides:* a startling discovery during screening colonoscopy. Case Rep gastroenterol.

[9] Pullan RL, Smith JL, Jasrasaria R, Brooker SJ (2014). Global numbers of infection and disease burden of soil transmitted helminthic in 2010. Parasit Vectors.

[10] Thompson RG (1988). Special veterinary pathology.

[11] Okewole EA, Oduye OO (2001). Experimental studies on hookworm infection. II. Hematological and plasma protein changes following *Ancylostoma caninum* infection in dogs. Tropical Vet.

[12] Pessoa LM, Morais SM, Bevilaqua CM, Luciano JHS (2002). Anthelmintic activity essential oil of *Ocimun gratissimum* Linn. and eugenol against *Haemonchus contortus*. Vet Parasitol.

[13] Vieira LS, Chagas ACS, Molento MB, Cavalcante ACR, Vieira LS, Chagas ACS, Molento MB (2009). Nematoides gastrintestinais e pulmonares de caprinos. Doenc¸ as Parasitárias de Caprinos e Ovinos-Epidemiologia e Controle.

[14] Charlier J, Höglund J, von Samson-Himmelstjerna G, Dorny P, Vercruysse J (2009). Gastrointestinal nematode infections in adult dairy cattle: impact on production, diagnosis and control. Vet Parasitol.

[15] Musella V, Catelan D, Rinaldi L, Lagazio C, Cringoli G, Biggeri A (2011). Covariate selection in multivariate spatial analysis of ovine parasitic infection. Preventive Vet Med.

[16] Sutherland IA, Leathwick DM (2011). Anthelmintic resistance in nematode parasites of cattle: a global issue?. Trends Parasitol.

[17] Melo ACFL, Reis IF, Bevilaqua CML, Vieira LS, Echevarria FAM, Melo LM (2003). Nematódeos resistentes a anti-helmíntico em rebanhos de ovinos e caprinos do estado do Ceará. Bras Ciência Rural.

[18] Kaplan RM (2004). Drug resistance in nematodes of veterinary importance. A status report. Trends Parasitol.

[19] Ekawardhani S, Anggoro UT, Krissanti I (2021). Anthelmintic potential of medicinal plants against *Ancylostoma caninum*. Vet Med Int.

[20] Agyare C, Spiegler V, Sarkodie H, Asase A, Liebau E, Hensel A (2014). An ethnopharmacological survey and *in vitro* confirmation of the ethnopharmacological use of medicinal plants as anthelmintic remedies in the Ashanti region, in the central part of Ghana. J Ethnopharmacol.

[21] Hoste H, Torres-Acosta JFJ, Sandoval-Castro CA, Mueller-Harvey I, Sotiraki S, Louvandini H (2015). Tannin containing legumes as a model for nutraceuticals against digestive parasites in livestock. Vet Parasitol.

[22] Pathak AK, Dutta N, Banerjee PS, Goswami TK, Sharma K (2016). Effect of condensed tannins supplementation through leaf meal mixture on voluntary feed intake, immune response and worm burden in *Haemonchus contortus* infected sheep. J Parasit Dis.

[23] Soetan KO, Lasisi OT, Agboluaje AK (2011). Comparative assessment of *in vitro* anthelmintic effects of the chloroform extracts of the seeds and leaves of the African locust bean (*Parkia biglobosa*) on bovine nematode eggs. J Cell and Anim Biol.

[24] Akhtar MS, Igbal Z, Khan MN, Lateef M (2000). Anthelmintic activity of medicinal plants with particular reference to their use in animals in the Indo-Pakistan subcontinent. Small Rumin Res.

[25] Alawa CBI, Adamu AM, Gefu JO, Ajanusi OJ, Abdu PA, Chiezey NP (2003). *In vitro* screening of Nigerian medicinal plants (*Vernonia amygdalina* and *Annona senegalensis*) for anthelmintic activity. Vet Parasitol.

[26] Carvalho CO, Chagas ACS, Cotinguiba F, Furlan M, Brito LG, Chaves FCM (2012). The anthelmintic effect of plant extracts on *Haemonchus contortus* and *Strongyloides venezuelensis*. Vet Parasitol.

[27] Ishola IO, Adeyemi OO, Agbaje EO, Tota S, Shukla R (2013). *Combretum mucronatum* and *Capparis thonningii* prevent scopolamine-induced memory deficit in mice. Pharm Biol.

[28] Betti JL, Lejoly J. Importance en médecine traditionnelle de *Combretum mucronatum* Schum. & Thon (Combretaceae) dans le Dja (Cameroun). In: Nasi R, Amsalem I, Drouineau S, editors. La gestion des forêts denses africaines aujourd’hui. Libreville, Séminaire FORAFRI, session 3: produits de la forêt. CD-Rom; 1999. p. 1–16.

[29] Koné WM, Vargas M, Keiser J (2011). Anthelmintic activity of medicinal plants used in Cote d’Ivoire for treating parasitic diseases. Parasitol Res.

[30] Spiegler V, Sendker J, Petereit F, Liebau E, Hensel A (2015). Bioassay-guided fractionation of a leaf extract from *Combretum mucronatum* with anthelmintic activity: oligomeric procyanidins as the active principle. Mol.

[31] Bele AA, Khale A (2011). An overview on thin layer chromatography. Int J Pharm Sci & Res.

[32] Orman E, Oppong Bekoe S, Asare-Nkansah S, Kralisch I, Jato J, Spiegler V (2023). Development of an analytical workflow to support the establishment of monographs in African Pharmacopoeias - *Combretum mucronatum* leaves as example. Planta Med.

[33] Raulf MK, Lepenies B, Strube C (2021). *Toxocara canis* and *Toxocara cati* somatic and excretory-secretory antigens are recognised by C-type lectin receptors. Pathog.

[34] Coles G, Jackson F, Pomroy WE, Prichard RK, Von Samson-Himmelstjerna G, Silvestre A (2006). The detection of anthelmintic resistance in nematodes of veterinary importance. Vet Parasitol.

[35] Vercruysse J, Holdsworth P, Letonja T, Barth D, Conder G, Hamamoto K (2001). International harmonisation of anthelmintic efficacy guidelines. Vet Parasitol.

[36] Demeler J, Kuttler U, von Samson-Himmelstjerna G (2010). Adaptation and evaluation of three different *in vitro* tests for the detection of resistance to anthelmintics in gastrointestinal nematodes of cattle. Vet Parasitol.

[37] Demeler J, Krucken J, AlGusbi S, Ramunke S, De Graef J, Kerboeuf D (2013). Potential contribution of P-glycoproteins to macrocyclic lactone resistance in the cattle parasitic nematode *Cooperia oncophora*. Mol Biochem Parasitol.

[38] Bekoe EO, Mireku-Gyimah NA, Boamah VE, Martinson S (2021). Phytochemical analysis and mycobactericidal studies of the leaves of *Combretum mucronatum* Schumach & Thonn. Int J Pharm Sci Res.

[39] Louis H, Akakuru OU, Linus MN, Innocent J, Pigweh IA (2018). Qualitative and quantitative phytochemical analysis of *Sclerocarya birrea* and *Sterculia setigera* in Kem and Yola, Adamawa State, Nigeria. Am J Biomed Res.

[40] Yongwa G, Belga FNFN, Ndjonka D, Saotoing P (2020). *In vitro* anthelmintic activity of aqueous and ethanolic extract of *Senna italitica* (Caesalpiniaceae) on three-stages of *Haemonchus contortus*. J Pharm Res Int.

[41] Hoste H, Torres-Acosta JF, Alonso-Diaz MA, Brunet S, Sandoval-Castro C, Adote SH (2008). Identification and validation of bioactive plants for the control of gastrointestinal nematodes in small ruminants. Tropical Biomed.

[42] Jato J, Waindok P, Belga FNFN, Orman E, Agyare C, Oppong Bekoe E (2023). Anthelmintic activities of extract and Ellagitannins from *Phyllanthus urinaria* against *Caenorhabditis elegans* and zoonotic or animal parasitic nematodes. Planta Med.

[43] Spiegler V, Liebau E, Peppler C, Raue K, Werne S, Strube C (2016). A hydroalcoholic extract from *Paullinia pinnata* L. roots exerts anthelmintic activity against Free-living and parasitic nematodes. Planta Med.

[44] Hounzangbe-Adote MS, Paolini V, Fouraste I, Moutairou K, Hoste H (2005). *In vitro* effects of four tropical plants on three life-cycle stages of the parasitic nematode, *Haemonchus contortus*. Res Vet Sci.

[45] Molan AL, Alexander RA, Brookes IM, McNabb WC (2000). Effects of an extract from sulla (*Hedysarum coronarium*) containing condensed tannins on the migration of three sheep gastrointestinal nematodes *in vitro*. Proc N Z Soc Anim Prod.

[46] Shepherd F, Chylinski C, Hutchings MR, Lima J, Davidson R, Kelly R (2022). Comparative analysis of the anthelmintic efficacy of European heather extracts on *Teladorsagia circumcincta* and *Trichostrongylus colubriformis* egg hatching and larval motility. Parasit Vectors.

[47] Barrau E, Fabre N, Fouraste I, Hoste H (2005). Effect of bioactive compounds from sainfoin (*Onobrychis viciifolia* Scop.) on the in vitro larval migration of *Haemonchus contortus*: role of tannins and flavonol glycosides. Parasitol.

[48] Greiffer L, Liebau E, Herrmann FC, Spiegler V (2022). Condensed tannins act as anthelmintics by increasing the rigidity of the nematode cuticle. Sci Rep.

[49] Klongsiriwet C, Quijada J, Williams AR, Mueller-Harvey I, Williamson EM, Hoste H (2015). Synergistic inhibition of *Haemonchus contortus* exsheathment by flavonoid monomers and condensed tannins. Int J Parasitol Drugs Drug Resist.

[50] Williams AR, Ropiak HM, Fryganas C, Desrues O, Mueller-Harvey I, Thamsborg SM (2014). Assessment of the anthelmintic activity of medicinal plant extracts and purified condensed tannins against free-living and parasitic stages of *Oesophagostomum dentatum*. Parasit Vectors.

[51] Scalbert A (1991). Antimicrobial properties of tannins. Phytochem.

[52] Quijada J, Fryganas C, Ropiak HM, Ramsay A, Mueller-Harvey I, Hoste H (2015). Anthelmintic activities against *Haemonchus contortus* or *Trichostrongylus colubriformis* from small ruminants are influenced by structural features of condensed tannins. J Agric and Food Chem.

[53] Martin RJ (1993). Neuromuscular transmission in nematode parasites and antinematodal drug action. Pharmacol Ther.

[54] Köhler P (2001). The biochemical basis of anthelmintic action and resistance. Int J Parasitol.

[55] Sasa MT, Alan PR, Cheryl LC, Richard JM (2002). Levamisole receptor phosphorylation: effects of kinase antagonists on membrane potential responses in *Ascaris suum* suggest that CaM kinase and tyrosine kinase regulate sensitivity to levamisole. J Exp Biol.

[56] Hagerman AE, Butler LG (1981). The specificity of proanthocyanidin-protein interactions. J Biol Chem.

[57] Hoste H, Martinez-Ortiz-De-Montellano C, Manolaraki F, Brunet S, Ojeda-Robertosa N, Fourquaux I (2012). Direct and indirect effects of bioactive tannin-rich tropical and temperate legumes against nematode infections. Vet Parasitol.

[58] Thompson DP, Geary TG, Marr JJ (1995). The structure and function of helminth surfaces. Biochemistry and molecular biology of parasites.

[59] Fetterer RH, Rhoads ML (1993). Biochemistry of the nematode cuticle: relevance to parasitic nematodes of livestock. Vet Parasitol.

[60] Fetterer RH (1989). The cuticular proteins from free-living and parasitic stages of Haemonchus contortus – I. Isolation and partial characterization. Comp Biochem Physiol.

[61] Vargas-Magaňa JJ, Torres-Acosta JFJ, Aguilar-Caballero AJ, Sandoval-Castro CA, Hoste H, Chan-Pérez JI (2014). Anthelmintic activity of acetone-water extracts against *Haemonchus contortus* eggs: interactions between tannins and other plant secondary compounds. Vet Parasitol.

[62] Chan-Pérez JI, Torres-Acosta JF, Sandoval-Castro A, Hoste H, Castaneda-Ramirez GS, Vilarem G (2016). *In vitro* susceptibility of ten *Haemonchus contortus* isolates from different geographical origins towards acetone: water extracts of two tannin rich plants. Vet Parasitol.

[63] de Fernex EVS, Alonso-Díaz MA, Mendoza de Gives P, Valles de la Mora B, González-Cortazar M, Zamilpa A (2015). Elucidation of *Leucaena leucocephala* anthelmintic-like phytochemicals and the ultrastructural damage generated to eggs of *Coop**eria spp*. Vet Parasitol.

[64] Novobilský A, Mueller-Harvey I, Thamsborg SM (2011). Condensed tannins act against cattle nematodes. Vet Parasitol.

[65] Heckendorn F, Häring DA, Maurer V, Zinsstag J, Langhans W, Hubertus HH (2006). Effect of sainfoin (*Onobrychis viciifolia*) silage and hay on established populations of *Haemonchus contortus* and *Cooperia curticei* in lambs. Vet Parasitol.

[66] Desrues O, Peña-Espinoza M, Hansen TVA, Enemark HL, Thamsborg SM (2016). Anti-parasitic activity of pelleted sainfoin (*Onobrychis viciifolia*) against *Ostertagia ostertagi* and *Cooperia oncophora* in calves. Parasit Vectors.

